# Molecular Tuning of IR-786 for Improved Tumor Imaging and Photothermal Therapy

**DOI:** 10.3390/pharmaceutics14030676

**Published:** 2022-03-19

**Authors:** Wonbong Lim, Jae Yong Byun, Gayoung Jo, Eun Jeong Kim, Min Ho Park, Hoon Hyun

**Affiliations:** 1 Department of Premedical Program, School of Medicine, Chosun University, Gwangju 61452, Korea; wonbong@chosun.ac.kr; 2 Madisarang Hospital, Cheongju 61469, Korea; bjy@madisarang.kr; 3 Department of Biomedical Sciences, Chonnam National University Medical School, Hwasun 58128, Korea; jky6213@naver.com; 4 Department of Surgery, Chonnam National University Medical School and Hwasun Hospital, Hwasun 58128, Korea; angeleunei@naver.com; 5 BioMedical Sciences Graduate Program (BMSGP), Chonnam National University, Hwasun 58128, Korea

**Keywords:** photothermal therapy, near-infrared fluorescence imaging, tumor targeting, near-infrared fluorophores, IR-786

## Abstract

A tumor-targeted near-infrared (NIR) fluorophore CA800Cl was developed based on commercially available IR-786 by modulating its physicochemical properties. IR-786, a hydrophobic cationic heptamethine cyanine fluorophore, was previously recognized as a mitochondria-targeting NIR agent with excellent optical properties. Owing to the poor tumor specificity of IR-786 itself, in vivo studies on tumor-targeted imaging have not yet been investigated. A chloro-cyclohexene ring and indolium side groups on the heptamethine chain are key structural features that improve tumor targetability, owing to better biodistribution and clearance. Thus, IR-786 should be designed to be more soluble in aqueous solutions so that it can preferentially accumulate in the tumor based on the structure-inherent targeting strategy. In this study, we developed a bifunctional NIR fluorophore CA800Cl by incorporating carboxylate moieties in the basic structure of IR-786. This improved its tumor targetability and water solubility, thereby enabling the use of CA800Cl for enhanced photothermal cancer therapy.

## 1. Introduction

Heptamethine cyanine dyes are a major class of near-infrared (NIR) fluorophores that have recently emerged as promising multifunctional agents for cancer targeting, imaging, and phototherapy [1,2,3]. A combination of NIR fluorophores, tumor-targeting ligands, and/or anticancer drugs is a typical strategy used to achieve simultaneous tumor-specific imaging and therapeutics [4,5]. Despite the significant progress in cancer theranostics, the delivery, specificity, and therapeutic potential of this strategy carries uncertainty because the targetability of ligands or drugs may be altered after chemical conjugation [1]. To mitigate this challenge, several heptamethine cyanine dyes, such as MHI-148 (also called IR-808), IR-783, and IR-780, have been reported to possess selectivity towards tumors compared with normal tissue without the need for chemical conjugation to tumor-specific ligands; these dyes have been proposed as a structure-inherent targeting strategy [6,7,8].

The tumor-targeting mechanism is yet to be fully understood; however, it is clear that the chloro-cyclohexene ring on the heptamethine skeleton is the key to improving tumor targetability. The preferential tumor uptake of heptamethine cyanine dyes is generally explained by the involvement of organic anion-transporting polypeptides (OATPs) [9,10]. Interestingly, Usama et al. recently demonstrated that a *meso*-chloride placed on the heptamethine bridge in serum can form a covalent adduct with albumin. This undergoes receptor-mediated endocytosis of albumin, resulting in an improved tumor accumulation through the enhanced permeability and retention effect [11,12]. Thus, the previously mentioned heptamethine cyanine dyes have been extensively used for in vivo tumor-targeted imaging and/or targeted drug delivery carriers after conjugation with non-selective drugs [5]. Owing to their tumor-targeting and NIR light-to-heat conversion properties, they have been further utilized as photothermal agents to deliver thermal energy directly to tumors after NIR light irradiation for effective photothermal therapy (PTT) [13,14,15]. However, the hydrophobic MHI-148 and IR-780 exhibit cytotoxic effects, which is a major bottleneck for many biological applications [1]. Although IR-783 has better water solubility and lower toxicity, its nonspecific uptake in normal tissues/organs and slow clearance from the body remain a challenge [1,16]. Therefore, it is necessary to develop functional NIR fluorophores with improved physicochemical properties that maintain tumor-targeting specificity but also possess good water solubility with optimal removal efficiency to reduce in vivo toxicity.

**Scheme 1:**
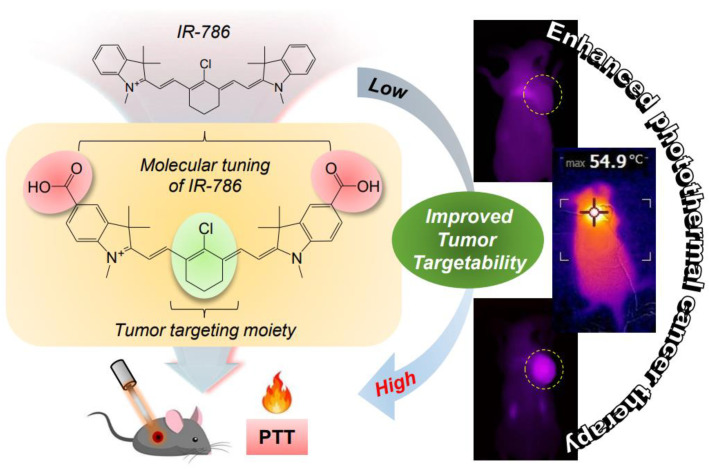
Schematic illustration of the molecular tuning of IR-786 for enhanced photothermal cancer therapy. The integration of a chloro-cyclohexene ring and side carboxyl groups on the heptamethine skeleton is the key to improving tumor targetability based on a structure-inherent targeting strategy.

IR-786, a hydrophobic cationic heptamethine cyanine fluorophore, was previously recognized as a mitochondria-targeting NIR agent with excellent optical properties [17]. Additionally, IR-786 was employed as a photosensitizer in vitro to encapsulate into nanocarriers for the poorly water-soluble IR-786, which is useful in photodynamic therapy [18]. Moreover, Choi et al. investigated the efficacy of IR-786–anticancer drug conjugates in vitro, which exhibited improved potency and specificity on patient-derived glioblastoma cell lines [19,20]. Owing to the poor tumor specificity of IR-786 itself, in vivo studies on tumor-targeted imaging have not yet been investigated. Therefore, IR-786 should be designed to be more soluble in aqueous solutions so that it can preferentially accumulate in the tumor based on the structure-inherent targeting strategy. In this study, we developed a bifunctional NIR fluorophore CA800Cl by incorporating carboxylate moieties in the basic structure of IR-786. This improved its tumor targetability and water solubility, thereby enabling the use of CA800Cl for enhanced photothermal cancer therapy (Scheme 1).

## 2. Materials and Methods

### 2.1. Synthesis of CA800Cl and CA800H NIR Fluorophores

All chemicals and solvents were purchased from Sigma-Aldrich (St. Louis, MO, USA) and used without further purification. Glacial acetic acid (15 mL) was added to a mixture of 4-hydrazinobenzoic acid **1** (1 g, 6.6 mmol) and 3-methyl-2-butanone **2** (1.1 mL, 9.9 mmol) in a round-bottomed flask fitted with a condenser. The brown suspension was refluxed for 8 h, and the solvent was removed under reduced pressure with a rotavapor. The residue was redissolved into a clear solution using water and methanol (10 mL, 90/10 *v*/*v*%). Undissolved material was filtered off, the filtrate was allowed to stand at room temperature, and the yellow crystal **3** (0.9 g, 67%) was collected by filtration. Iodomethane (1.22 mL, 3.8 mmol) followed by acetonitrile (15 mL) were added to a dried flask containing carboxylated indole **3** (0.5 g, 2.5 mmol), and the suspension was refluxed for 18 h. The precipitate intermediate **4** was collected by filtration and washed with acetonitrile and ethyl acetate. The collected solid was used directly in the next step without further purification (0.6 g, 70%). A mixture of heterocyclic salt **4** (0.1 g, 0.29 mmol), Vilsmeier–Haack reagent **5** (0.037 g, 0.13 mmol) for CA800H or Vilsmeier–Haack reagent **6** (0.046 g, 0.13 mmol) for CA800Cl, and anhydrous sodium acetate (0.04 g, 0.48 mmol) in absolute ethanol (5 mL) was heated under reflux for 6 h, respectively. The reaction mixtures were cooled to ambient temperature, and then dried by rotovap to remove the reaction solvent, and purified as dark-green powders (CA800H; 0.04 g, 62% and CA800Cl; 0.05 g, 67%). The final products were separated using a preparative high-performance liquid chromatography (HPLC) system (Waters, Milford, MA, USA). The molecular weights of the purified samples were confirmed by Dionex UltiMate^TM^ 3000 mass spectrometry system (Thermo Scientific, Waltham, MA, USA).

### 2.2. Optical and Physicochemical Property Analyses

All optical measurements were performed in phosphate-buffered saline (PBS) at pH 7.4. The absorption spectra of NIR fluorophores were recorded using a fiber optic FLAME absorbance and fluorescence (200–1025 nm) spectrometer (Ocean Optics, Dunedin, FL, USA). The molar extinction coefficient was calculated using the Beer–Lambert equation. To determine the fluorescence quantum yield (*Φ*), indocyanine green (ICG) dissolved in dimethyl sulfoxide (DMSO) (*Φ* = 13%) was used as a calibration standard under the conditions of matched absorbance at 770 nm [4]. The fluorescence emission spectra of each NIR fluorophore were analyzed using a SPARK^®^ 10M microplate reader (Tecan, Männedorf, Switzerland) at excitation wavelengths ranging from 700 to 760 nm and emission wavelengths ranging from 750 to 900 nm. In silico calculations of the partition coefficient (log*D* at pH 7.4) and the topological polar surface area (TPSA) were conducted using Marvin and JChem calculator plugins (ChemAxon, Budapest, Hungary).

### 2.3. In Vitro Photothermal Conversion Efficiency

Based on the equation reported previously [21,22], the photothermal conversion efficiency (*η*) of the CA800Cl NIR fluorophore was calculated as follows:

$$\eta =\frac{hA\Delta T_{max}-Q_{s}}{I\left(1-10^{-A_{\lambda }}\right)}$$

where *h* is the heat transfer coefficient, *A* is the surface area of the container, ΔT_max_ is the temperature change of the sample solution at the maximum temperature, *I* is the laser power density, *A_λ_* is the absorbance of sample at 808 nm, and Q*_s_* is the heat associated with the light absorbance of the solvent.

### 2.4. In Vitro Cancer Cell Binding and NIR Fluorescence Microscopy

Human large-cell lung carcinoma cell line NCI-H460 was obtained from the American Type Culture Collection (ATCC, Manassas, VA, USA). Cancer cells were maintained in Roswell Park Memorial Institute (RPMI) 1640 medium (Gibco BRL, Paisley, UK) supplemented with 10% fetal bovine serum (FBS, Gibco BRL) and an antibiotic–antimycotic solution (Welgene, Daegu, South Korea) in a humidified 5% CO_2_ atmosphere at 37 °C. When the cells reached a confluence of approximately 50%, they were rinsed twice with PBS, and each NIR fluorophore was added to each well at various concentrations in the range of 2–20 μM. The cells were incubated for 24 h at 37 °C and then washed with PBS. NIR fluorescence imaging was performed using a Nikon Eclipse Ti-U inverted microscope system (Nikon, Seoul, South Korea).

### 2.5. In Vitro Cytotoxicity Assay

Cell toxicity and proliferation were confirmed using a 3-(4,5-dimethylthiazol-2-yl)-2,5-diphenyltetrazolium bromide (MTT, Sigma-Aldrich) assay. Mouse embryonic fibroblast cell line NIH/3T3 was obtained from the ATCC and maintained in Dulbecco’s Modified Eagle Medium (DMEM) medium (Gibco BRL) supplemented with a 10% FBS and an antibiotic–antimycotic solution (Welgene) in a humidified 5% CO_2_ atmosphere at 37 °C. NIH/3T3 cells were seeded onto 96-well plates (1 × 10^4^ cells per well). To evaluate the cytotoxicity as a function of the concentration, the cells were treated with each NIR fluorophore (2, 10, 20, and 50 μM) for 1 h and cultured for 24 h after treatment. At each assay time point, the incubation cell medium was replaced with 100 μL of fresh medium, and 10 μL of the MTT solution was directly added to each 100 μL well. Subsequently, the plates were incubated for 4 h at 37 °C in a humidified 5% CO_2_ incubator. Finally, the 96-well plates were placed in a microplate reader (SPARK^®^ 10M, Tecan) to measure the absorption intensity at 570 nm. Cell viability was calculated using the following formula: cell viability (%) = (*A*_sample_ − *A*_blank_)/(*A*_control_ − *A*_blank_) × 100, where *A* is the average absorbance.

### 2.6. Serum Protein Binding Assay

A mixture of bovine serum albumin (BSA, MW ≈ 67 kDa; 0.15 µmol, 10 mg) and CA800Cl (0.2 µmol, 0.11 mg) in PBS (1 mL) was incubated at 37 °C for 4 h. The mixture was separated using a gel-filtration chromatography (GFC) system with Econo-Pac P6 cartridges (Bio-Rad, Hercules, CA, USA) and a flow rate of 1 mL/min (PBS, pH 7.4). The hydrodynamic diameter of BSA complex was measured at 280 nm using the ÄKTAprime plus system (GE Healthcare, Piscataway, NJ, USA). The separated BSA & CA800Cl complex was analyzed by an absorbance spectrometer (Ocean Optics).

### 2.7. NCI-H460 Xenograft Mouse Model

Animal protocols were conducted in accordance with the guidelines approved by Chonnam National University Animal Research Committee (CNU IACUC-H-2020-19). Adult (6 weeks old, ≈25 g) male NCRNU mice were purchased from OrientBio (Seongnam, South Korea). NCI-H460 cancer cells were cultured and suspended in 100 μL of PBS before they were subcutaneously inoculated in the right flank of each mouse (1 × 10^6^ cells per mouse). When tumor sizes reached approximately 1 cm in diameter, each NIR fluorophore was administered intravenously. The animals were euthanized for in vivo NIR fluorescence imaging within a designated period of time.

### 2.8. In Vivo Biodistribution and Tumor Imaging

In vivo NIR fluorescence imaging was performed using an FOBI imaging system (NeoScience, Suwon, South Korea). Mice were sacrificed at 24 h post-injection of each NIR fluorophore, and their main organs and tumors were harvested and imaged to confirm the time-dependent biodistribution of each NIR fluorophore. The fluorescence intensities on the tumor sites and excised organs were determined using the open source ImageJ software (National Institutes of Health, Bethesda, MD, USA).

### 2.9. In Vivo Photothermal Therapeutic Efficacy

CA800Cl or PBS were intravenously injected into the NCI-H460 tumor-bearing mice and the mice were anaesthetized after 24 h. The tumors were treated with a laser (1.1 W/cm^2^, *λ* = 808 nm) for 5 min. Temperature changes at the tumor sites were monitored using a thermal imager (FLIR Systems, Wilsonville, OR, USA). Tumors were resected from each group 24 h after irradiation for subsequent histological observation after completing the hematoxylin and eosin (H&E) staining procedure. To assess the in vivo antitumor effect, the macroscopic tumor growth of each group was monitored for 9 days. The tumor volume (V) was measured by the following formula: V = 0.5 × longest diameter × (shortest diameter)^2^.

### 2.10. Statistical Analysis

Statistical analysis was performed by one-way analysis of variance for multiple comparison test. The results were represented as mean ± standard deviation (S.D.). A value of *p* < 0.05 was considered statistically significant.

### 2.11. Histological Analysis

Tumors resected from each group were preserved for H&E staining and microscopic observation. The tumor samples were fixed in 4% paraformaldehyde and stored in a deep freezer. Frozen tumors were cryosectioned (10 µm-thick), stained with H&E, and observed using a microscope. Histological analysis was performed on a Nikon Eclipse Ti-U inverted microscope system (Nikon).

## 3. Results and Discussion

### 3.1. Synthesis and Characterization of NIR Fluorophores

**Figure 1:**
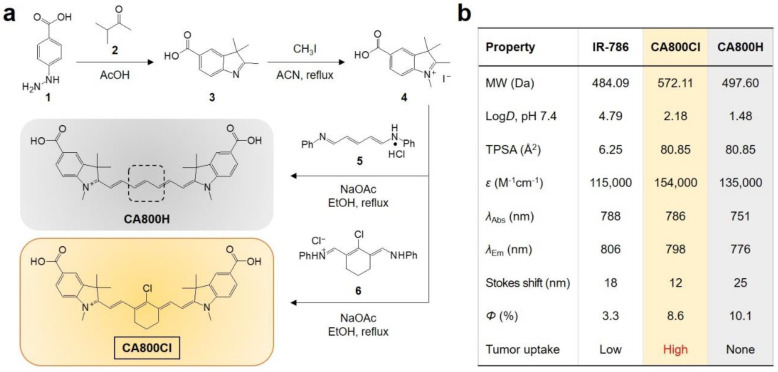
(**a**) Synthetic scheme of the CA800Cl and CA800H NIR fluorophores. (**b**) Physicochemical and optical properties of IR-786, CA800Cl, and CA800H NIR fluorophores. In silico calculations of the partition coefficient (log*D* at pH 7.4) and TPSA were calculated using Marvin and JChem calculator plugins (ChemAxon).

With an aim to improve the tumor targetability and hydrophilicity of IR-786, the carboxylated form of IR-786, namely CA800Cl, was designed to synthesize for the first time. More importantly, two carboxyl groups attached to both sides of the IR-786 structure were optimally selected from the several hydrophilic groups such as hydroxyl, methoxy, amine, sulfonate, and carboxyl groups after the in silico prediction of their log*D* values (–OH, 5.42; –OMe, 4.47; –NH_2_, 3.13; –SO_3_H, 2.80; and –COOH, 2.18). As shown in Figure 1a, the symmetrical heptamethine cyanine fluorophore CA800Cl was synthesized by the condensation reaction between carboxylated indolium (**4**) and the Vilsmeier–Haack reagent (**6**) in the presence of anhydrous sodium acetate. An additional type of heptamethine cyanine fluorophore, namely CA800H, was prepared using another type of the Vilsmeier–Haack reagent (**5**) under the same conditions. The structure of CA800H is similar to CA800Cl, except for the absence of a chloro-cyclohexene ring on the heptamethine cyanine backbone. The chloro-cyclohexene ring of heptamethine cyanines plays a key role in OATPs- or albumin-mediated tumor uptake; however, the tumor-targeting domain of CA800Cl can be identified by comparing it with that of CA800H to support the theory of targeting.

**Figure 2:**
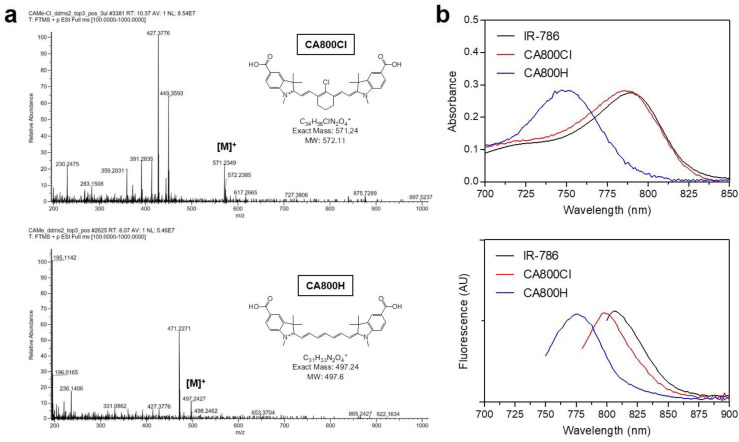
(**a**) Mass spectra of CA800Cl and CA800H NIR fluorophores. (**b**) Absorption spectra and fluorescence emission spectra of IR-786, CA800Cl, and CA800H NIR fluorophores. Optical measurements were performed in PBS, pH 7.4.

As shown in Figure 2a, the successful syntheses of CA800Cl and CA800H were confirmed via mass spectrometry before proceeding with the next steps. The absorption and emission peaks of IR-786, CA800Cl, and CA800H were all in the NIR region (700–900 nm) and they exhibited 12–25 nm Stokes shifts (Figure 2b). As summarized in Figure 1b, carboxylated CA800Cl exhibited a higher hydrophilicity (log*D* = 2.18), polarity (TPSA = 80.85 Å^2^), molar extinction coefficient (*ε* = 154,000 M^−1^cm^−1^), and quantum yield (*Φ* = 8.6%) than that of IR-786 (4.79, 6.25 Å^2^, 115,000 M^−1^cm^−1^, and 3.3%, respectively) measured in PBS at pH 7.4. This indicates that the improved hydrophilicity of CA800Cl may contribute to its enhanced optical properties and, furthermore, tumor targetability.

### 3.2. In Vitro Cytotoxicity and Cancer Cell Binding

**Figure 3:**
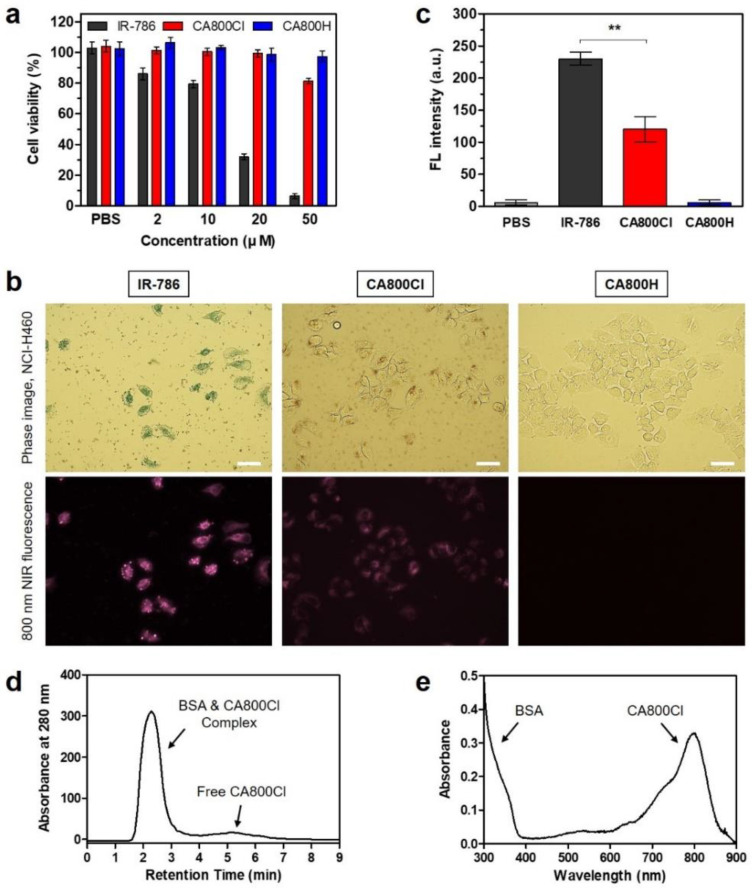
(**a**) Cell viability assay of each NIR fluorophore using NIH/3T3 cells. Percentage cytotoxicity is determined after 24 h of treatment with various concentrations of each NIR fluorophore. (**b**) Live cancer cell binding and (**c**) relative fluorescence intensity of each NIR fluorophore in NCI-H460 cancer cells. Phase contrast and NIR fluorescence images of cell line are obtained using 10 µM of each NIR fluorophore. Data are expressed as mean ± S.D. (*n* = 3). ** *p* < 0.01. All NIR fluorescence images have identical exposure times and normalization. Scale bar = 100 μm. (**d**) GFC separation of a mixture of BSA and CA800Cl. The hydrodynamic diameter of BSA complex was measured at 280 nm using an absorbance spectrophotometer. (**e**) Absorption spectrum of BSA & CA800Cl complex. The sample was obtained from the first peak fraction as shown in (**d**).

An in vitro cell viability test of NIH/3T3 cells was conducted using the MTT assay after the cells were incubated with various concentrations of IR-786, CA800Cl, and CA800H for 24 h (Figure 3a). No obvious cytotoxicity of CA800Cl and CA800H was observed between 0 and 20 μM. However, the cell viability in IR-786 test group decreased considerably with an increase in concentration. This result demonstrates that CA800Cl has good biocompatibility, owing to its improved water solubility. Moreover, we observed the intracellular distribution of each NIR fluorophore after 24 h of incubation in NCI-H460 cells (Figure 3b). Interestingly, IR-786 and CA800Cl exhibited significant intracellular localization with specific fluorescence signals in the cytoplasm, owing to the presence of the chloro-cyclohexene ring. CA800H displayed no cellular uptake under the same condition, owing to the absence of the chloro-cyclohexene ring, which formed a covalent adduct with albumin for endocytosis. However, it remains unclear if CA800Cl exhibited a relatively weak fluorescence intensity in the cells compared with that of IR-786 (Figure 3c). Although IR-786 is more favorable to bind the cancer cells for in vitro conditions, it shows low tumor targetability for in vivo conditions because of its high nonspecific uptake in various tissues and organs.

Additionally, we investigated the serum protein binding of CA800Cl mixed with BSA to identify whether CA800Cl could be targeted to the tumor by albumin-mediated endocytosis in vivo. Serum protein binding was measured by GFC system after incubating the CA800Cl in BSA solution at 37 °C for 4 h (Figure 3d). The separated BSA complex was then collected from the first peak fraction and confirmed by the absorption peaks of BSA and CA800Cl, respectively (Figure 3e). This indicates that the meso-chloride on a rigid cyclohexenyl ring of CA800Cl is responsible for the formation of covalent albumin adducts trapped in tumors via the albumin-mediated endocytosis [11,12].

### 3.3. Time-Dependent In Vivo Tumor Imaging and Biodistribution

**Figure 4:**
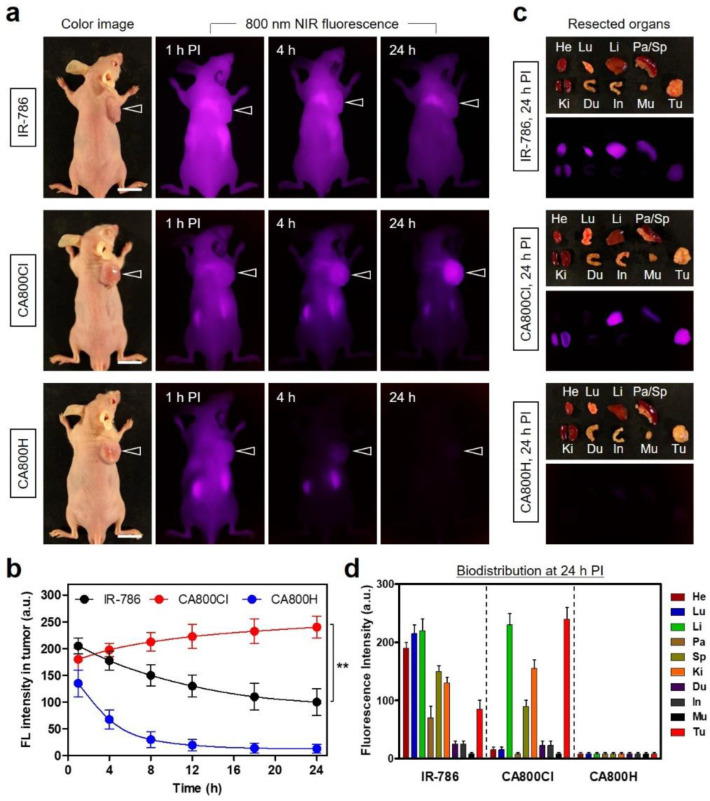
In vivo NCI-H460 tumor targeting efficiency and biodistribution of NIR fluorophores. (**a**) NIR fluorescence imaging at 1 h, 4 h, and 24 h post-injection of IR-786, CA800Cl, and CA800H, respectively. (**b**) Time-dependent fluorescence intensities at the tumor sites targeted by each NIR fluorophore. (**c**) Resected organs and tumors imaged 24 h after injection of each NIR fluorophore. (**d**) Quantitative fluorescence analysis of intraoperative dissected organs 24 h post-injection of each NIR fluorophore. The tumor site is indicated by an arrowhead. Abbreviations: Du, duodenum; He, heart; In, intestine; Ki, kidneys; Li, liver; Lu, lungs; Mu, muscle; Pa, pancreas; Sp, spleen; Tu, tumor; and PI, post-injection. Scale bars = 1 cm. All NIR fluorescence images have identical exposures and normalizations. Data are expressed as mean ± S.D. (n = 3). ** *p* < 0.01.

After confirming the superior biocompatibility of CA800Cl in vitro, the tumor targetability of CA800Cl in vivo was investigated in the NCI-H460 xenograft tumor model. Then, 10 nmol of IR-786, CA800Cl, or CA800H was intravenously injected into tumor-bearing mice, and NIR fluorescence imaging was subsequently performed for 24 h post-injection in real-time (Figure 4a). Results from the time-dependent NIR fluorescence imaging revealed that the fluorescence intensity at the tumor site treated with CA800Cl continuously increased for 24 h after administration, whereas the fluorescence signal from the tumor injected with IR-786 gradually decreased without peak accumulation until 24 h post-injection (Figure 4b). Notably, the tumors exhibited no significant uptake of CA800H within 4 h after injection. This result reconfirms that the chloro-cyclohexene ring on the heptamethine cyanine backbone of IR-786 and CA800Cl plays a critical role in tumor accumulation. Moreover, the higher tumor accumulation of CA800Cl than that of IR-786 may be due to the improved hydrophilicity, which may prolong the blood circulation time. We further investigated the biodistribution and clearance of each NIR fluorophore 24 h after injection (Figure 4c). As expected, IR-786 exhibited high nonspecific uptake in the heart, lungs, liver, and other major organs in the body 24 h post-injection. CA800Cl showed high fluorescence only in the liver and kidneys, which was similar to the tumor tissue, thereby resulting in improved distribution and clearance from the organs (Figure 4d). Interestingly, CA800H did not exhibit any nonspecific tissue/organ uptake 24 h after injection. This indicates that the carboxyl groups of the CA800Cl structure may help to reduce the background fluorescence caused by nonspecific uptake, especially in regions adjacent to the tumor. Therefore, the carboxylated form of IR-786, i.e., CA800Cl, is of particular importance because it presents: (i) better water solubility and biocompatibility, (ii) lower nonspecific uptake within 24 h post-injection, and (iii) higher tumor targetability for improved tumor imaging and PTT.

### 3.4. In Vitro and In Vivo Photothermal Effects

**Figure 5:**
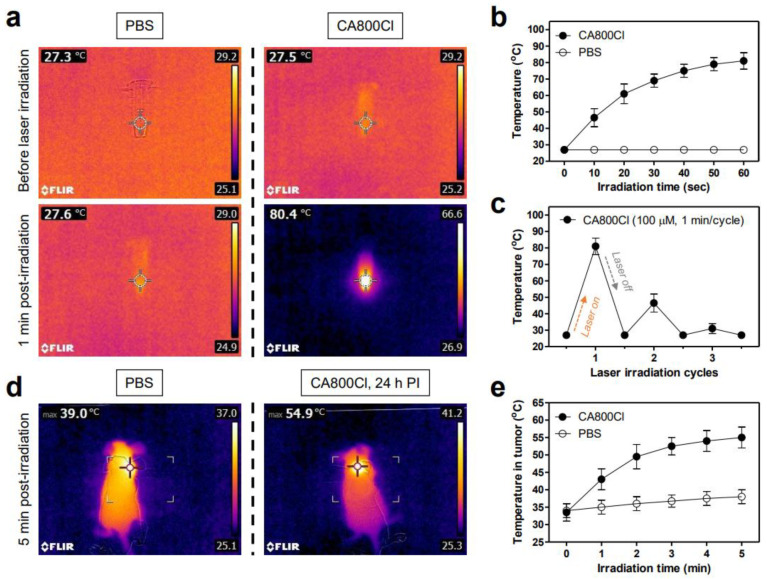
(**a**) In vitro photothermal images of CA800Cl solution (100 μM in PBS) and PBS alone (100 μL) exposed to an 808 nm laser (1.1 W/cm^2^) for 1 min. The maximum temperature was automatically recorded using an infrared thermal camera as a function of irradiation time. (**b**) Temperature changes in the solutions in each sample were monitored during the 1 min of laser irradiation. (**c**) Photothermal stability curve of 100 μM CA800Cl solutions after repeated laser irradiation (1 cycle = 1 min of laser irradiation). (**d**) Whole-body photothermal images of tumor-bearing mice 24 h post-injection of PBS or CA800Cl upon exposure to 808 nm laser irradiation (1.1 W/cm^2^) for 5 min. (**e**) Temperature changes at the tumor sites in each treatment group were monitored during the 5 min of 808 nm laser irradiation. Data are expressed as mean ± S.D. (n = 3).

To examine the photothermal effect of CA800Cl, we monitored temperature variations in solutions of CA800Cl (100 μM in PBS) and PBS alone irradiated by an 808 nm NIR laser (1.1 W/cm^2^) for 1 min. The temperature changes were monitored in real-time using a thermal imager. The temperature of the CA800Cl solution remarkably increased from 27.5 to 80.4 °C under laser irradiation for 1 min, whereas the PBS alone displayed no temperature change under the same conditions (Figure 5a). The temperature of the CA800Cl solution rapidly reached approximately 70 °C within the first 30 s of laser irradiation and was maintained up to 80 °C for the next 30 s of irradiation (Figure 5b). Based on the equation described in the Materials and Methods section, the photothermal conversion efficiency (*η*) of CA800Cl was calculated to be 30.2%, which is comparable to that of the heptamethine cyanine dyes that were reported previously [21,22]. This suggests that CA800Cl can be used as an effective PTT agent for in vivo photothermal tumor ablation. Additionally, three cycles of laser irradiation were repeatedly performed using the CA800Cl solution to evaluate its photothermal stability (Figure 5c). As expected, the temperature of the CA800Cl solutions rapidly decreased in the second cycle and was mostly minimized in the third cycle under repeated laser irradiation. This indicates that the polymethine cyanine structure can easily be destroyed by photobleaching during exposure to concentrated NIR light. Thus, it is essential to improve the tumor targetability of cyanine-based PTT agents for efficient tumor ablation.

Furthermore, the in vivo PTT capability of CA800Cl was investigated using an NCI-H460 tumor-bearing mouse model. Mice were intravenously administered with CA800Cl (10 nmol, 0.23 mg/kg) or PBS 24 h before laser irradiation and exposed to an 808 nm laser (1.1 W/cm^2^) for 5 min. Previously, the power density of the 808 nm laser was optimally adjusted to prevent unnecessary damage in normal tissue owing to laser power alone, without any PTT agent [23,24]. The temperature of the tumors treated with CA800Cl markedly increased up to 54.9 °C after 5 min of laser irradiation, whereas tumor temperatures in the PBS-injected mice exhibited minimal change (39 °C) under the same condition (Figure 5d). Notably, tumor temperatures in the CA800Cl-treatment group reached approximately 50 °C after 2 min of laser irradiation and were maintained at 55 °C for the next 3 min of laser irradiation, which was sufficient to induce tumor necrosis (Figure 5e). This result demonstrates that CA800Cl can be successfully used as a tumor-targetable PTT agent for complete tumor ablation.

### 3.5. In Vivo Photothermal Therapeutic Efficacy

**Figure 6:**
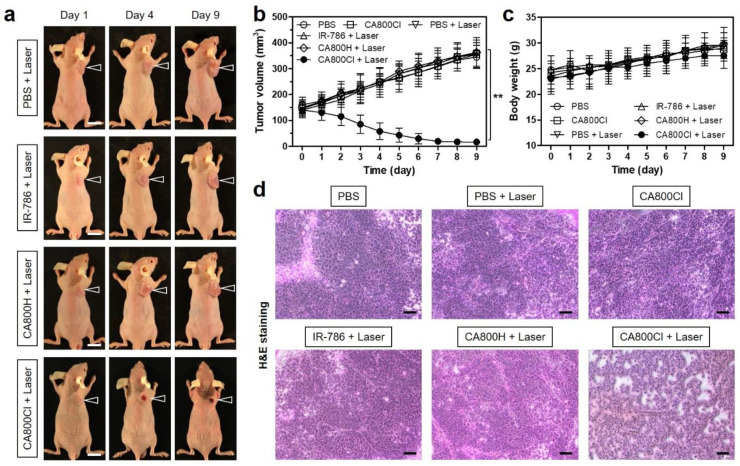
In vivo phototherapeutic efficacy of CA800Cl NIR fluorophore. (**a**) Representative photos of changes in tumor size in NCI-H460 tumor-bearing mice for 9 days after different treatments. The laser groups were treated with 24 h post-injections of PBS, IR-786, CA800H, and CA800Cl, followed by 808 nm laser irradiation (1.1 W/cm^2^) for 5 min. Scale bars = 1 cm. (**b**) Tumor growth rates and (**c**) body weights of each treatment group were monitored for 9 days. Data are expressed as mean ± S.D. (n = 3). ** *p* < 0.01. (**d**) Tumor sections stained with H&E from each group 24 h after different treatments. Scale bars = 100 μm.

To further evaluate the phototherapeutic effects of CA800Cl, NCI-H460 tumor-bearing mice were periodically monitored after different PTT treatments (Figure 6a). The tumor growth in each group was measured for 9 days to assess the suppression and recurrence of tumors. The tumor volumes in the CA800Cl-injected group gradually decreased within 5 days after PTT, and, finally, only black scars remained on the original tumor sites. Without the sufficient tumor accumulation, the tumors treated with IR-786 or CA800H followed by laser irradiation displayed similar growth rates to the control group treated with PBS alone and laser irradiation, which indicates that there were no phototherapeutic effects and tissue damage caused by laser irradiation alone (Figure 6b).

The body weights of the mice in all groups were measured during the treatment period (Figure 6c). The CA800Cl-treated group exhibited no distinct change in body weight compared with other groups, which indicates a favorable tolerance of the phototherapy and good in vivo biocompatibility of CA800Cl. Moreover, the phototherapeutic efficacy in each group after different PTT treatments was evaluated using histological tissue images via H&E staining (Figure 6d). Distinct apoptotic and necrotic signs of the tumor tissues were observed in the CA800Cl treatment group upon laser irradiation, whereas no change in the cancer cell sizes, shapes, or damage was observed in the treatment groups using PBS and CA800Cl alone, or PBS, IR-786 and CA800H with laser irradiation. This demonstrates that cancer cells can be efficiently destroyed by the photothermal energy generated from the combination of CA800Cl and NIR laser irradiation.

## 4. Conclusions

In summary, we have successfully developed a new type of NIR fluorophore, CA800Cl, based on the molecular tuning of IR-786 to improve both water solubility and tumor targetability for a structure-inherent cancer targeting approach. CA800Cl exhibited higher optical properties and biocompatibility as well as improved in vivo performance. Furthermore, the tumor-targetable CA800Cl enabled the production of thermal energy after NIR laser irradiation, which demonstrated its applicability to fluorescence-guided cancer phototherapy. In particular, the molecular design approach in this study may provide inspiration for optimization of designs of conventional heptamethine cyanine dyes. Therefore, the single NIR fluorophore CA800Cl may hold great potential as a multifunctional theranostic agent for simultaneous cancer targeting, imaging, and phototherapy.

## Data Availability

Not applicable.
